# Supplementary material for: Inferring the Joint Demographic History of Multiple Populations from Multidimensional SNP Frequency Data
Source: PLoS Genet. 2009 Oct 23;5(10):e1000695. doi: 10.1371/journal.pgen.1000695 (PMC2760211; doi:10.1371/journal.pgen.1000695)
Supplement: Text S1 — Complete supplementary data. (1.80 MB PDF) [file pgen.1000695.s001.pdf]

Text S1

Inferring the Joint Demographic History ofMultiple Populations from Multidimensional SNP Frequency Data

Ryan N. Gutenkunst, Ryan D. Hernandez,
Scott H. Williamson, & Carlos D. Bustamante

Contents

1 Properties of the AFS **2**
1.1 Distinguishing reduced divergence and increased migration 2

2 Numerical methods **3**
2.1 Finite-difference scheme 3
2.2 Population splits 4
2.3 Non-uniform grid 5
2.4 Extrapolation 5
2.5 Small-M comparison with *ms* 6

3 Data processing **7**
3.1 Projection 7
3.2 LD calculation 8
3.3 Estimating μ 8

4 Coalescent simulations **8**
4.1 Reduction of effective independent SNPs by linkage 9

5 Out of Africa model **9**
5.1 Marginal analyses 9
5.2 Maximum likelihood parameters 10
5.3 Projection and residuals 11
5.4 Parametric bootstrap 12
5.4.1 Parameter correlations 12
5.4.2 Sloppiness 16
5.5 Contemporary migration test 16
5.6 Rare alleles 17

6 Settlement of New World model **19**
6.1 Alternative demographic models . . . 19
6.2 Maximum likelihood parameters . . . 20
6.3 Parametric bootstrap . . . 20
6.3.1 Parameter correlations . . . 21
6.4 Comparison with Out of Africa model parameters . . . 21
6.5 Admixture variability . . . 21
6.6 Rare alleles . . . 23

7 Nonsynonymous variation **28**

1 Properties of the AFS

1.1 Distinguishing reduced divergence and increased migration

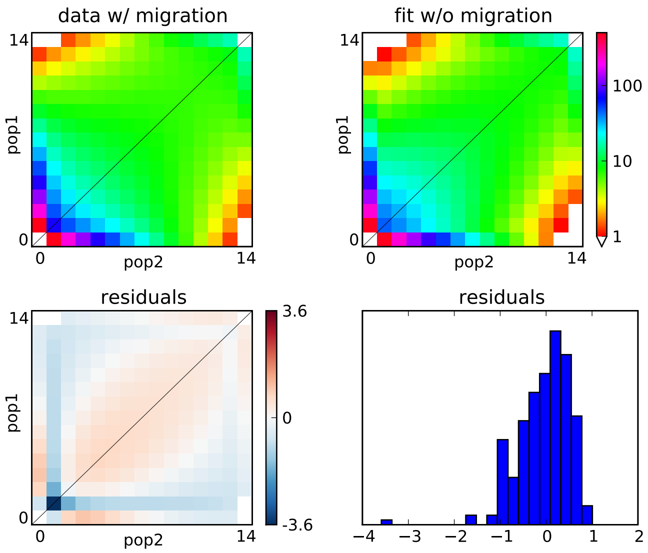

Supplementary Figure 1: Fitting data including migration with a no-migration model.

As an example of inference with migration, consider the demographic scenario of Figure 1 for parameter values of moderate migration and divergence: $\theta = 1000$, $\nu_1 = \nu_2 = 0.5$, $M = 2$, and $\tau = 0.3$. The noise-free AFS is shown in the upper left panel of Supplementary Figure 1. For a model with $M \equiv 0$ fit to this AFS, the maximum likelihood parameters are: $\theta = 1034.4$, $\nu_1 = \nu_2 = 0.44$, and $\tau = 0.084$. As expected, when we neglect migration the inferred divergence time is substantially smaller. Less obviously, the ancestral population size (proportional to θ) is overestimated, while current populations sizes are underestimated. The resulting AFS is shown in the upper right panel of Supplementary Figure 1 and is

qualitatively very similar to the AFS with migration. As illustrated in the lower left panel, however, fitting this incorrect model yields correlated residuals; the model predicts too few shared polymorphisms at low frequency in one or both populations. This is the AFS signal that distinguishes between reduced divergence time and increased migration.

2 Numerical methods

2.1 Finite-difference scheme

Note: here we index populations using lower-case Greek indices, to distinguish from the numerical index we use over grid points.

To numerically approximate the solution to our diffusion equation (Eqn. 1) for P populations, we discretize the equation over a non-uniform regular P-dimensional grid. (The grid is described in section 2.3). To solve this multidimensional problem, we adopt an alternating difference scheme [1], in which we evolve terms involving derivatives in each variable x_α separately. Here we outline the finite difference scheme we use, inspired by work of Chang and Cooper [2] on diffusion equations arising in plasma physics.

Importantly, aside from the boundary conditions our diffusive evolution conserves probability. For stability and accuracy our finite difference scheme should also explicitly conserve probability. This motivates us to consider the *fluxes* F_α. Our diffusion equation can be decomposed into fluxes as

$$\frac{\partial \phi}{\partial \tau} = \frac{1}{2}\sum_\alpha \frac{\partial^2}{\partial^2 x_\alpha}\left(V^{(\alpha)}\phi\right) - \sum_\alpha \frac{\partial}{\partial x_\alpha}\left(M^{(\alpha)}\phi\right) \tag{S1}$$

$$= \sum_\alpha \frac{\partial}{\partial x_\alpha}\left[\frac{1}{2}\frac{\partial}{\partial x}\left(V^{(\alpha)}\phi\right) - M^{(\alpha)}\phi\right] \tag{S2}$$

$$= \sum_\alpha \frac{\partial}{\partial x_\alpha} F_\alpha, \tag{S3}$$

where $V^{(\alpha)} = x_\alpha(1-x_\alpha)$, and $M^{(\alpha)} = \sum_\beta M_{\alpha\leftarrow\beta}(x_\beta - x_\alpha)$.

If we take a centered finite difference about point j on our grid x_i, where $i = 1, 2, \ldots G$, we obtain

$$\frac{\phi_j^{t+1} - \phi_j^t}{\Delta t} = \frac{1}{\Delta_j}\left(F^{t+1}_{j+1/2} - F^{t+1}_{j-1/2}\right). \tag{S4}$$

To conserve total probability (as defined by the trapezoidal numerical integration scheme) we take $F_{-1/2} \equiv F_{G+1/2} \equiv 0$ and

$$\Delta_j = \begin{cases} (x_1 - x_0)/2, & j = 0, \\ (x_{i+1} - x_{i-1})/2, & 0 < j < G, \\ (x_G - x_{G-1})/2, & j = G. \end{cases} \tag{S5}$$

To evaluate $F^{t+1}_{j+1/2}$, we take a centered finite-difference, so that

$$F^{t+1}_{j+1/2} \equiv \frac{1}{x_{j+1}-x_j}\frac{1}{2}\left(V_{j+1}\,\phi^{t+1}_{j+1} - V_j\,\phi^{t+1}_j\right) - M_{j+1/2}\,\phi^{t+1}_{j+1/2} \tag{S6}$$

where $\phi^{t+1}_{j+1/2} \equiv \frac{1}{2}\left(\phi^{t+1}_j + \phi^{t+1}_{j+1}\right)$. (Note that Chang and Cooper take a non-centered difference on ϕ to ensure non-negativity of the equilibrium solution. This is not possible for us, because the equilibrium solution of our equations can be singular.)

Combining equations S4 and S6 yields a tridiagonal system of equations which can be solved by standard methods, e.g. $a_j\phi^{t+1}_{j-1} + b_j\phi^{t+1}_j + c_j\phi^{t+1}_{j+1} = \phi^t_j$.

The flux out is handled by particular absorbing terms [3] of the form:

$$\frac{\phi^{t+1}_0 - \phi^t_0}{\Delta t} = \frac{1}{\Delta_0}\left(-\frac{1}{2}\left.\frac{\partial V}{\partial x}\right|_{x=0} + M_0\right)\phi^{t+1}_0, \tag{S7}$$

$$\frac{\phi^{t+1}_G - \phi^t_G}{\Delta t} = \frac{1}{\Delta_G}\left(\frac{1}{2}\left.\frac{\partial V}{\partial x}\right|_{x=1} - M_G\right)\phi^{t+1}_G. \tag{S8}$$

These are applied only at the grid points where all frequencies equal 0 or 1 respectively.

New mutations are injected into the system at each timestep via:

$$\frac{\phi^{t+1}_1 - \phi^t_1}{\Delta t} = \frac{\theta}{2}\frac{1}{x_1}\frac{2^P}{(x_2-x_0)x_1^{P-1}} \tag{S9}$$

The final term (involving 2^P) normalizes the probability density given trapezoid rule integration over the other dimensions of the grid. Note that we inject probability at a rate $\theta/(2x_1)$, independent of the relative population size ν_α. This reflects the fact that mutations are being input at x_1, not $1/(2N_\alpha)$. The effect of relative population size is accounted for by the drift term V.

In total, for a P-dimensional problem, each timestep requires solving G^{P-1} tridiagonal systems of size G. We set our timestep to be $\Delta t \equiv \min \Delta x/10$, which provides good stability and accuracy for the scenarios considered here. (Situations with very high migration rates or selection coefficients or very small population sizes may require finer timesteps.) Thus the total run time for a single likelihood evaluation is order G^{P+1}.

2.2 Population splits

Numerically handling δ-function density for ϕ upon population splits (Eqn. 2) involves some subtlety. In particular, it is important to properly conserve ϕ density, and the value z to which a given pair of x and y map to may not be a point in the grid.

Consider x and y which map to a value z which lies between grid points z_i and z_{i+1}. Define $\Delta z_i \equiv z_{i+1} - z_i$. We distribute the density $\phi(x,y)$ which a proportion a going to z_i and b going to z_{i+1}, such that

$$a = \frac{z_{i+1}-z}{\Delta z_i}, \qquad b = \frac{z - z_i}{\Delta z_i}. \tag{S10}$$

To ensure proper normalization under the trapezoid rule in the interior of our domain, we must have

$$\frac{a}{2}C\Delta z_{i-1} + \frac{a+b}{2}C\Delta z_i + \frac{b}{2}C\Delta z_{i+1} = \phi(x,y). \tag{S11}$$

Upon splitting, a contribution aC is thus added to element $\phi(x,y,z_i)$ and a contribution bC to $\phi(x,y,z_{i+1})$.

The edge cases, in which z, for example, maps between z_1 and z_2 are handled similarly. In those cases, Eqn. S11 must simply be altered to reflect the correct trapezoidal integration rule for those edge cases.

2.3 Non-uniform grid

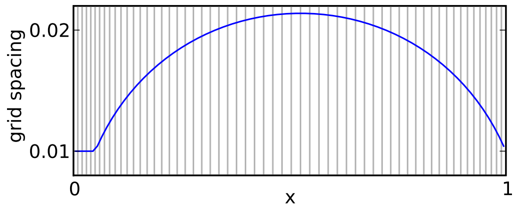

Supplementary Figure 2: The nonuniform grid used in our numerical solution of the diffusion Eqn. 1, for $G = 60$. The vertical lines denote grid points spaced on $(0, 1)$, while the blue line traces the spacing between adjacent grid points.

We solve our equations on a non-uniform x grid. For a grid of G points, the first $G/10$ points (rounded down) are uniformly spaced within $x \in [0, 0.05]$. This uniform grid spacing makes the solution more accurate in the low-frequency regime, where we have many segregating mutations and the data are therefore relatively less noisy.

The remaining points are placed so that their spacings increase quadratically, being finer at small and large values of x. This scheme minimizes the difference in spacings between adjacent grid points, which we found helped accuracy and stability. Specifically, we take the remaining points onto $q \in [0, 1]$ which is mapped onto x by $x = a\,q^3 + b\,q^2 + c\,q + d$, were $d = 0.05$, c is equal to the spacing between the first $G/10$ points, $b = -3(\Delta q + c + \Delta q\, d)/\Delta q$, $a = -(2/3)b$. This pattern of grid points is illustrated in Supplementary Figure 2.

Because our algorithm run time scales as $O(G^4)$ when working with 3 populations, this non-uniform grid gives a dramatic increase in computational speed for a given solution accuracy.

2.4 Extrapolation

Our numerical approximation to the solution of the diffusion equation improves as the grid becomes finer, as $G \to \infty$. Our computational costs, however, increase rapidly with G. To overcome this, we use Richardson extrapolation [1], extrapolating on the log of each entry

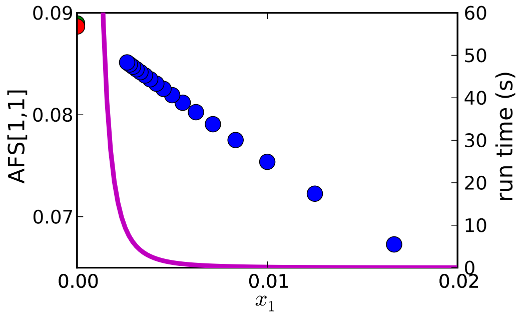

Supplementary Figure 3: Extrapolation results. The blue points show the evaluation of AFS[1,1] for $G = \{40, 50, ...200\}$. The green dot is the value extrapolated from the evaluations with $G = \{40, 50, 60\}$. The red dot is the result of averaging 10^8 simulations from `ms`. The purple line shows the run time for this two-dimensional problem as a function of grid spacing. The extrapolated evaluation took ≈0.5 seconds.

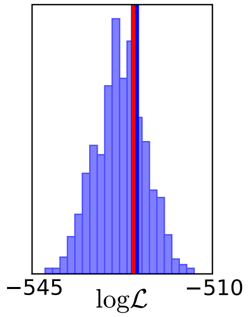

Supplementary Figure 4: Diffusion-coalescent comparison for low migration. As in Figure 1.E. from the main text, but with $M = 0.1$.

of the AFS, as in Eqn. 5. As illustrated in Supplementary Figure 3, this yields a massive improvement in the accuracy of our calculated AFS. (A more sophisticated treatment of the mutation influx boundary conditions [4] may alleviate some of the bias introduced by the finite grid, but extrapolation would still enhance accuracy.)

The demographic scenario simulated in Supplementary Figure 3 is as in Figure 1, with $\theta = 1$, $\nu_1 = \nu_2 = 1$, $M = 1$, $T = 0.5$. The corresponding *ms* command is:

```
ms 40 1e8 -t 1 -I 2 20 20 -n 1 1 -n 2 1 -ma x 2 2 x -ej 0.25 2 1 -en 0.25 1 1
```

2.5 Small-M comparison with *ms*

The human applications we consider here involve relatively high migration rates, so there are very few fixed differences between populations. In other cases, much smaller migration

rates may be of interest, so we have additionally compared performance between diffusion and coalescent simulations for $M = 0.1$, using the same procedure as reported in the main text for $M = 2$. Supplementary Figure 4 shows that our method is again very accurate in likelihood evaluation. The speed advantage of the diffusion method is similar in this case to the $M = 2$ case.

3 Data processing

The 219 genes we consider yield 5,017,161 bases of non-coding sequenced DNA, with 31,352 observed SNPs in the EGP samples. Table 8 gives a gene-by-gene break down of the data.

To apply the statistical correction for ancestral misidentification [5], we align to the panTro2 build of the chimp genome using BLAT [6]. Genes longer than the 25 kb recommended for BLAT were split into 25 kb chunks with 2 kb overlap. Each 25 kb chunk was required to have at least 90% identity with chimp across at least 70% of the sequence, or it was discarded from analysis.

To apply the ancestral misidentification correction to a SNP it must satisfy 4 criteria. 1) It must have been successfully aligned to chimp. 2) The chimp allele must be one of the segregating human alleles. 3) The two bases flanking the SNP must be the same in chimp and human. 4) The SNP cannot be adjacent to any other SNP. All these criteria are satisfied for 27,824 SNPs in our sample.

3.1 Projection

To account for missing data and ease visual comparison between populations, we project each AFS down to 20 samples per population using a hypergeometric distribution. In essence, to project from n successful calls in a population to m, we average over all possible results of choosing a size m subsample from those n calls. Because the sampling in each population is independent of sampling in the others, this is a simple extension of the one-dimensional case [7]. In particular, if we are projecting an AFS S from a sample size in population α of n to a sample size of m, the projected AFS P is

$$P[i_\alpha, i_\beta, \dots] = \sum_{d=i_\alpha}^{n-m+i_\alpha} \frac{\binom{m}{i_\alpha}\binom{n-m}{d-i_\alpha}}{\binom{n}{d}} S[d, i_\beta, \dots]. \tag{S12}$$

Note that this projection reduces the number of segregating SNPs, as SNPs segregating in the original sample may project to make contributions to the 'absent in the sample' and 'fixed in the sample' classes.

For our Out of Africa model, which uses YRI, CEU & CHB data, 25,258 SNPs have ≥ 20 calls in every population. We correct our estimation of the ancestral population size for the SNPs lost to the ancestral misidentification criteria and the ≥ 20 calls criteria by using an effective sequenced length. In this case, it is 5.02 Mb $\cdot\, 25,259/31,352 = 4.02$ Mb. After projection, our AFS sums to 17,446 segregating SNPs.

For the New World model we have 26,387 SNPs with ≥ 20 calls in every population, yielding an effective sequencing length of 4.20 Mb. Our projected AFS, however, contains only 13,290 segregating SNPs, reflecting lower diversity in these populations.

3.2 LD calculation

Figure 2.D. and Figure 3.D. show linkage disequilibrium as a function of physical distance in the data. These values were calculated using phased haplotypes generated using PHASE 2.0 [8] and provided by the EGP. For comparison with our simulated data, only SNPs in non-coding regions and with minor allele frequency $\geq 10\%$ in all populations were considered.

3.3 Estimating μ

We estimate the neutral mutation rate μ using the divergence between human and chimp. Comparing aligned sequences in our data, we estimate the divergence to be 1.13%. Assuming a divergence time of 6 million years [9] and a mean generation time for human and chimp over this interval of 25 years, we have

$$\mu = 0.0113 \cdot 25/(2 \cdot 6 \times 10^6) = 2.35 \times 10^{-8} \text{ per generation.} \tag{S13}$$

Note that the assumed generation time formally cancels in the conversion between genetic and chronological time units. If t_C is the chronological time, τ is the genetic time (in units of $2N_A$ generations), and t_g is the generation time, then

$$t_C = t_G \cdot t_g = \tau \cdot 2N_A \cdot t_g = \tau \cdot \frac{\theta}{2\mu} \cdot t_g. \tag{S14}$$

As shown in the previous paragraph, μ is proportional to t_g, resulting in the cancellation of t_g.

4 Coalescent simulations

Detailed simulations of the EGP data are required to estimate accurate confidence intervals and to perform realistic goodness-of-fit and hypothesis tests. For these simulations, we use *ms* [10].

To account for potential linkage between genes, in our simulations we divide the 219 sequenced genes into 194 potentially linked regions, each separated by at least 500 kb (Table 8). We simulate the entirety of each region using a value for θ scaled by the relative length of that region to the total sequenced length. The recombination rate for each region is set to the average recombination rate from the HapMap Release 22 genetic map [11]. This rate r was converted into a population-scaled rate $\rho = 2N_{ref}r$ by using N_{ref} inferred from θ and our estimate of μ.

Simulations use the total number of samples from each EGP population. The resulting AFS is then projected down to 20 samples per population. When extracting an AFS from a simulation, only SNPs lying within regions sequenced by the EGP are included.

4.1 Reduction of effective independent SNPs by linkage

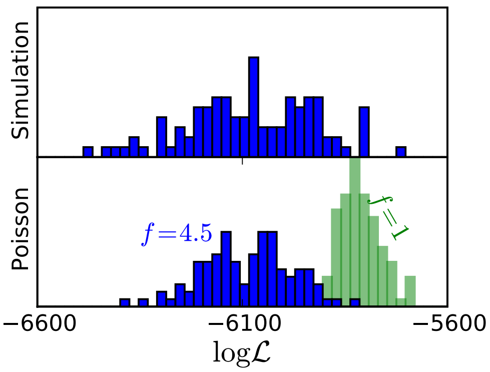

Supplementary Figure 5: Shown are histograms of the likelihoods of generated data sets under the model. Data sets in the top panel are simulated using the coalescent procedure described above. Data sets in the bottom panel are simulated using the modified Poisson procedure described below, where the effective number of data points has been reduced by a factor f.

To first order, the effect of linkage on our analysis is to reduce the effective number of independent samples in the frequency spectrum. To estimate the magnitude of this effect, in Supplementary Figure 5 we compare the distribution of likelihoods generated by the full simulation procedure described above and by modified Poisson sampling from the model frequency spectrum M. In the modified Poisson sampling, we sample from M/f and then multiply the resulting sampled frequency spectrum by f. In effect, this generates a frequency spectrum where each sample is replicated f times.

As illustrated in Supplementary Figure 5, the likelihood distributions are similar for $f = 4.5$. This suggests that linkage reduces the effective number of independent samples in our data by a factor of approximately 4.5. Note that the likelihood distribution for the full simulations remains somewhat wider than that for $f = 4.5$, indicating that the effect of the linkage in the data is more than a simple consistent reduction in the number of effective data points.

5 Out of Africa model

5.1 Marginal analyses

Supplementary Figure 6 and Supplementary Table 1 present results from a two-epoch model fit to the YRI spectrum. The fit is quite good, and the inferred parameter values are reasonable and consistent with estimates from other studies.

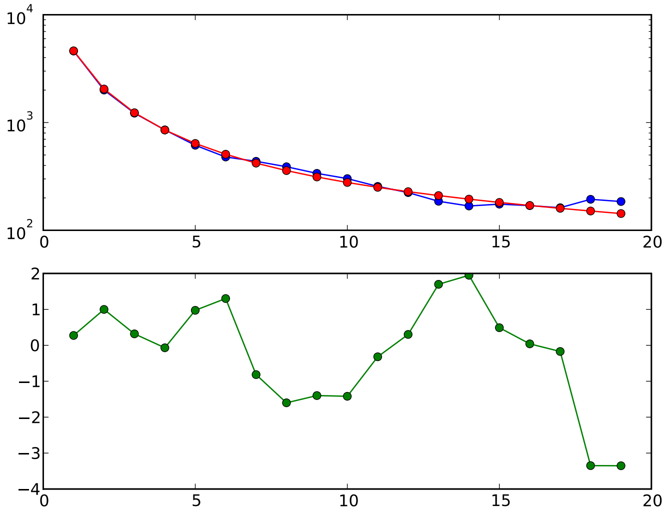

Supplementary Figure 6: YRI marginal fit. The top panel shows the model (red) and data (blue) spectra. The bottom panel shows the residuals.

Supplementary Table 1: Maximum-likelihood parameters for YRI marginal fit.

parameter	value
N_A	7,100
N_{AF}	13,600
T_{AF} (kya)	136

Supplementary Figure 7 and Supplementary Table 2 present results from a divergence and growth model fit to the CEU/CHB spectrum. (In this model both populations diverge from an equilibrium population and grow exponentially following a concurrent bottleneck.) Here we do see a pattern of correlated residuals, in that the model underestimates the amount of high frequency shared polymorphism. As seen in Figure 2, the more complex model which incorporates migration from YRI alleviates this somewhat. Allowing the bottleneck times in the two populations to occur anytime after divergence yielded only a very slight increase in fit quality (data not shown).

5.2 Maximum likelihood parameters

In genetic units (scaled by N_A), the maximum likelihood parameters are shown in Supplementary Table 3. In *ms* syntax, the demographic model is:

```
-n 1 1.682020 -n 2 3.736830 -n 3 7.292050
-eg 0 2 116.010723 -eg 0 3 160.246047
-ma x 0.881098 0.561966 0.881098 x 2.797460 0.561966 2.797460 x
```

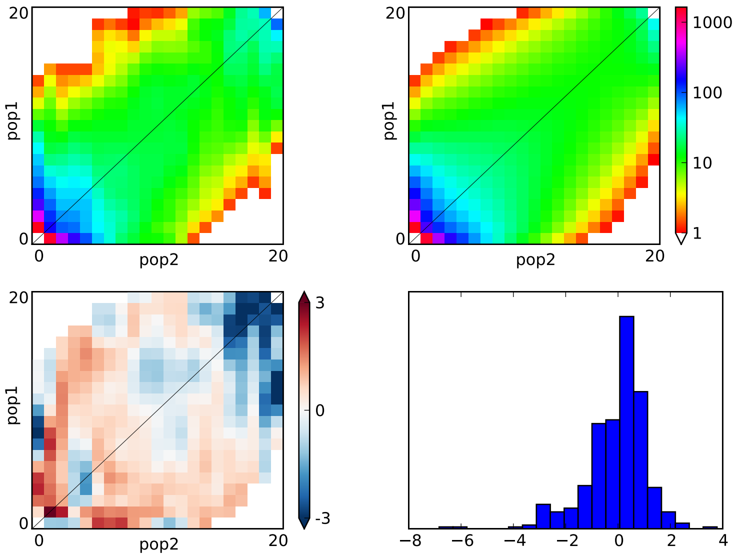

Supplementary Figure 7: CEU/CHB marginal fit. The upper left panel shows the data, and the upper right the model. The lower panels show the residuals, as a heat map and as a histogram.

```
-ej 0.028985 3 2 -en 0.028985 2 0.287184
-ema 0.028985 3 x 7.293140 x 7.293140 x x x x x
-ej 0.197963 2 1 -en 0.303501 1 1'
```

To convert to physical units, we use $\theta = 4N_A\mu L$, where L is the length of sequence considered. Importantly, L must use the effective sequenced length, which accounts for losses in alignment and missed calls. So

$$N_{ref} = \frac{\theta}{4L\mu} = \frac{2788.2}{4 \cdot 4.04 \times 10^6 \cdot 2.35 \times 10^{-8}} = 7310. \quad \text{(S15)}$$

The remaining conversions are straightforward.

5.3 Projection and residuals

Some the correlation between residuals seen in Figure 2 and 3 is due to the fact that we've projected the data down from a larger sample size. This effect is illustrated in Supplementary Figure 8. In subfigure (a) we show the residuals between our model AFS simulated for the total number of individuals in the EGP and a Poisson sampling from that AFS. As expected, the residuals shown no correlation. Subfigure (b) compares the model AFS with the same sampled AFS as in (a), but projected down to 20 samples per population. This projection results in correlation between adjacent entries in the AFS that is very similar to that seen in the model comparison with the real data.

Supplementary Table 2: Maximum-likelihood parameters for CEU/CHB marginal fit.

parameter	value
N_A	6,650
N_{EU0}	1,730
N_{EU}	20,000
N_{AS0}	330
N_{AS}	35,000
m_{EU-AS} ($\times 10^{-5}$)	34
T_{EU-AS} (kya)	18.4

Supplementary Table 3: Maximum likelihood parameter values for Out of Africa model, in genetic units.

parameter	value	parameter	value
θ_T	2788.2	M_{AF-B}	3.65
ν_{AF}	1.68	M_{AF-EU}	0.44
ν_B	0.287	M_{AF-AS}	0.28
ν_{EU0}	0.129	M_{EU-AS}	1.40
ν_{EU}	3.74	T_{AF}	0.607
ν_{AS0}	0.070	T_B	0.396
ν_{AS}	7.29	T_{EU-AS}	0.058

5.4 Parametric bootstrap

Shown in Supplementary Figure 9 are the results of the parametric bootstrap analysis of parameter uncertainties for our Out of Africa model. All distributions of bootstrap parameter estimates have their mode approximately at the maximum likelihood value. This provides empirical evidence that, as expected, our estimation is not significantly biased.

5.4.1 Parameter correlations

The correlations between parameter values inferred during our conventional bootstrap shed light on the dependencies between model parameters. Supplementary Figure 10 shows the squared correlation coefficient between each of the inferred parameter values. Correlations are typically low, with a few exceptions.

Supplementary Figure 11 illustrates three of the most strongly correlated parameter pairs. The strongest correlation is between the time of the African population size change T_{AF} and the ancestral population size N_A. This likely reflects the need to generate the appropriate level of polymorphism before divergence from Africa. Interestingly, the divergence time between CEU and CHB, T_{EU-AS}, is more strongly correlated with the growth rate of the Asian population r_{AS} than with the migration rate between the two populations m_{EU-AS}.

The lower-right panel of Supplementary Figure 11 compares inferred values of the initial population sizes for European and Asian populations, N_{EU0} and N_{AS0}, respectively. There is

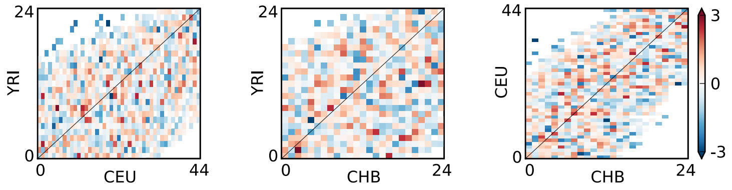

(a) Model comparison with sampled spectrum

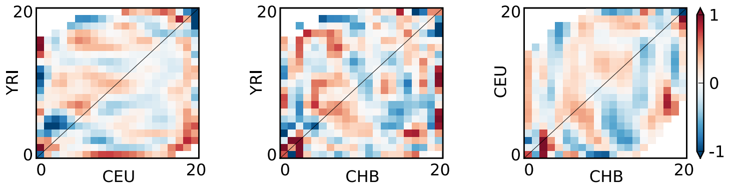

(b) Model comparison with projected spectrum

Supplementary Figure 8: Effect of projection on residuals. As shown in (b), projecting a data set down from a larger sample size yields correlated 'splotchy' residuals.

little correlation between the inferred values. 92 of our 100 bootstrap fits yield $N_{EU0} > N_{AS0}$.

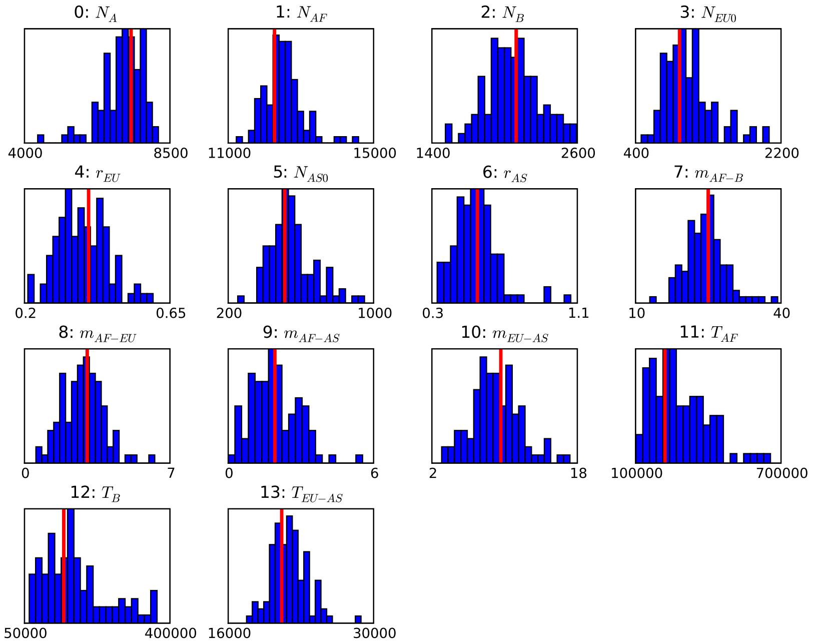

(a)

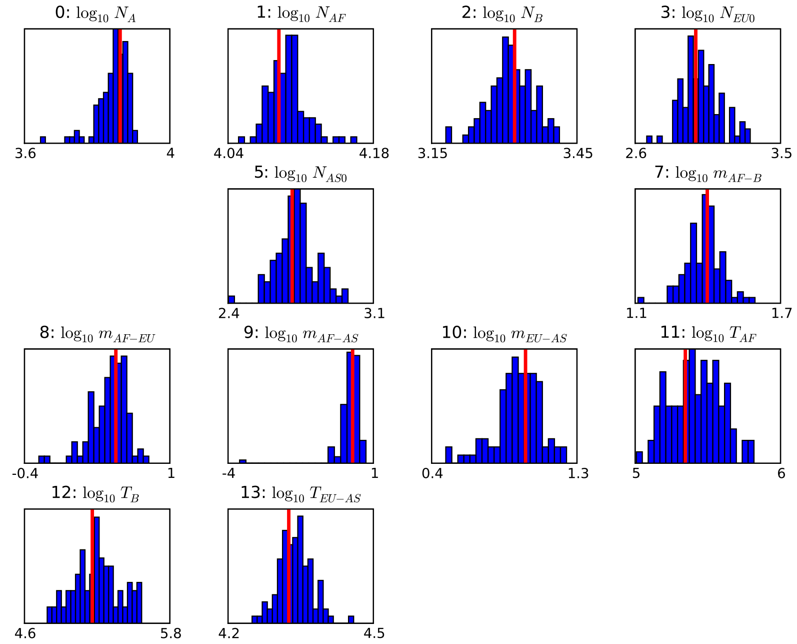

(b)

Supplementary Figure 9: Out of Africa model parametric bootstrap results. Shown the results of fits to 100 simulated data sets, generated using maximum likelihood values from the real data (red lines). For parameters defined to be positive, also shown are the logarithms of the bootstrap results, as these were used in confidence interval calculations. Particularly for T_{AF} and T_B, they are much closer to normally distributed.

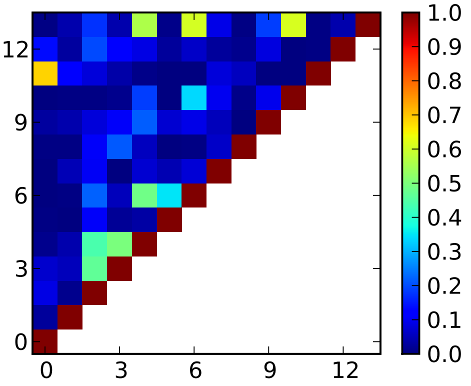

Supplementary Figure 10: Correlation (r^2) between bootstrap parameter values for Out of Africa analysis. Parameter indices are as in Supplementary Figure 9.

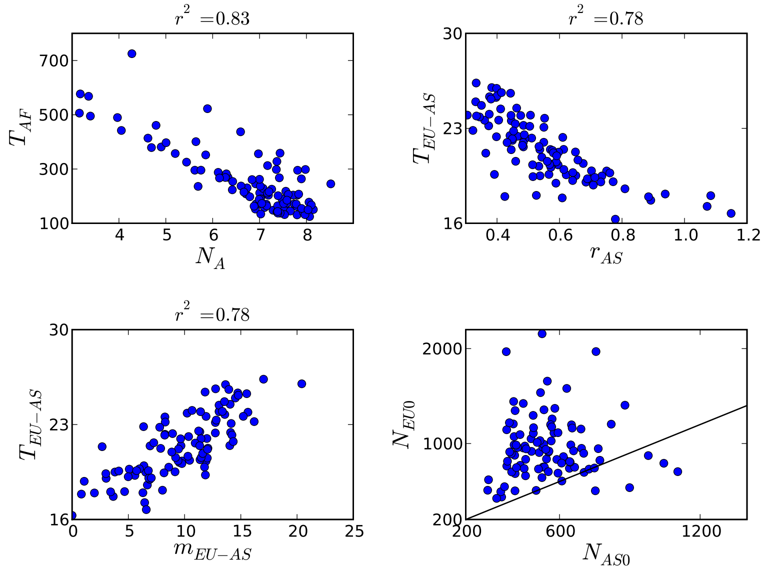

Supplementary Figure 11: The first three panels are the highly correlated pairs of parameters in our Out of Africa analysis. (N_A and T_{EU-AS} have been divided by 1000 for plotting, and m_{EU-AS} has been multiplied by 10^5.) The line in the lower right panel is $N_{EU0} = N_{AS0}$.

5.4.2 Sloppiness

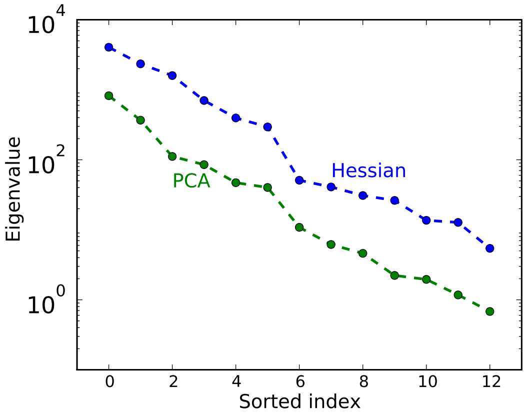

Supplementary Figure 12: Plotted are the eigenvalues of the model Hessian matrix and a Principle-Components-Analysis of our 100 parametric bootstrap parameter sets. The fact that these plots are roughly straight lines indicates that the model is 'sloppy'.

Recently it has been suggested that the sensitivities of many-parameter models to changes in those parameter values follow a universal 'sloppy' distribution [12]. This distribution is characterized by eigenvalues of the Hessian matrix $(\partial^2 \mathcal{L}/\partial \log p_i \partial \log p_j)$ which decrease linearly in their logarithm. As illustrated in Supplementary Figure 12, the demographic model we fit exhibits a sloppy spectrum of sensitivities. As expected, a principle components analysis of our ensemble of 100 parameter sets derived from fitting simulated data sets shows a similar sloppy pattern.

The sloppiness we see is indicative of strong correlations between the effects of different parameters on the AFS. In some problems, it is possible to define an orthogonal parameterization which eliminates these correlations [12]. Such a parameterization has been demonstrated for the single-population AFS [13], but none is know yet for the multiple-population case.

Additionally, the fact that this model is sloppy is interesting because the model was designed to be parsimonious, and in all previous examples of sloppy systems the parameterization was determined by biological or physical considerations. This example expands the domain of 'sloppy' problems.

5.5 Contemporary migration test

Fitting the data with a model lacking contemporary migration yields the parameters in Supplementary Table 4. In our test, we generate simulated data sets with these parameters, then fit them with a model allowing contemporary migration.

Supplementary Table 4: Maximum likelihood parameter values for Out of Africa model without contemporary migration, in genetic units.

parameter	value	parameter	value
θ_T	2532.4	M_{AF-B}	3.65
ν_{AF}	1.82		
ν_B	0.319		
ν_{EU0}	0.149		
ν_{EU}	8.41	T_{AF}	1.28
ν_{AS0}	0.066	T_B	0.897
ν_{AS}	34.36	T_{EU-AS}	0.052

5.6 Rare alleles

In Figure 2.E. of the main text, we evaluate the goodness-of-fit of our model to the observed data using our composite likelihood function. This function involves the entirety of the frequency spectrum, and in some cases specific frequency classes may be of particular interest. In particular, rare alleles may be of medical interest. Supplementary Table 5 compares our the rare-allele entries of our bootstrap simulations with the real data. Each entry in the tables records the fraction of bootstrap simulations which yielded a larger proportion of SNP in that entry that in the real data. For example, 3% of simulations yield a larger proportion of SNPs that are at frequency (2,1,1) in (YRI,CEU,CHB) samples, respectively, than is observed in the real data. In general, the proportions seen in the real data are typical in our simulations. For some entries the real data may be atypical of the simulations, but it is unclear whether these deviations are signficant.

Supplementary Table 5: P-values of rare frequency spectrum entries. The tables record the fraction of parametric bootstrap simulations yielding larger proportion of mutations in a given frequency class than observed in the data.

CEU				
2	0.45	0.24	0.34	
1	0.87	0.77	0.93	
0	—	0.68	0.35	
	0	1	2	CHB

(a) Sample YRI frequency 0

CEU				
2	0.38	0.22	0.49	
1	0.41	0.05	0.63	
0	0.17	0.82	0.63	
	0	1	2	CHB

(b) Sample YRI frequency 1

CEU				
2	0.51	0.05	0.28	
1	0.11	0.03	0.01	
0	0.65	0.50	0.13	
	0	1	2	CHB

(c) Sample YRI frequency 2

6 Settlement of New World model

6.1 Alternative demographic models

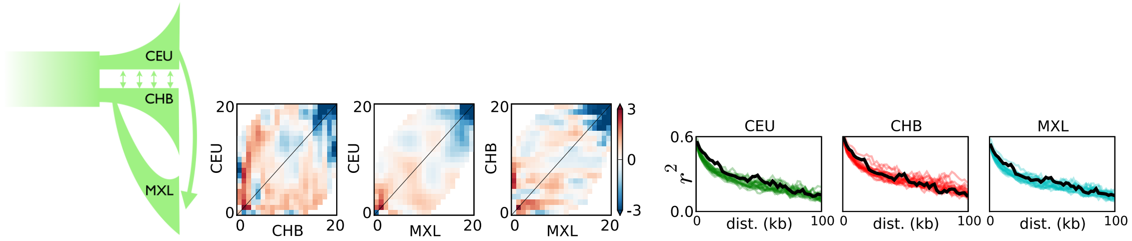

(a) Divergence from equilibrium population. $\log \mathcal{L} = -5405.9$, 11 free parameters

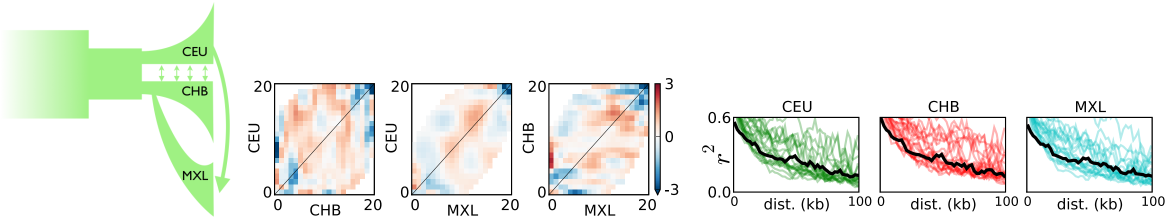

(b) Divergence from two-epoch population. $\log \mathcal{L} = -5261.8$, 13 free parameters

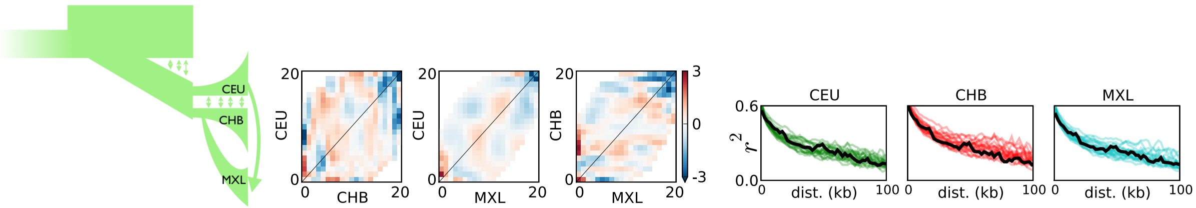

(c) With African population. $\log \mathcal{L} = -5262.0$, 10 free parameters

Supplementary Figure 13: Alternative models for joint CEU,CHB,MXL demographic history. Each panel shows one of the three models we considered for modeling settlement of the New World, which differ only in history prior to the MXL divergence. Each panel also shows the residuals from the fit to the data, along with a comparison of LD decay from the real data and from the simulations.

Supplementary Figure 13 illustrates several alternative demographic models considered for the Settlement of the New World. In Model (a) the CEU and CHB populations diverge from an equilibrium neutral population. Unsurprisingly, this model fails to reproduce the observed frequency spectrum well (log-likelihood = -5405.9, 11 free parameters). As an alternative, we considered Model (b), which allows for a population size change before divergence. (We allowed for the population to increase or decrease. The optimization resulting in a decrease.) This model reproduces the frequency spectrum substantially better (log-likelihood = -5261.1, 13 free parameters), but the predicted pattern of linkage disequilibrium is very different from the observed pattern. The model we settled upon is Model (c), in which the

Supplementary Table 6: Maximum likelihood parameter values for Settlement of the New World model, in genetic units.

parameter	value	parameter	value
ν_{EU0}	0.208	M_{EU-AS}	1.98
ν_{EU}	2.42	T_{EU-AS}	0.072
ν_{AS0}	0.081	T_{MX}	0.059
ν_{AS}	4.186	f_{MX}	0.48
ν_{MX0}	0.103		
ν_{MX}	7.94		

population history prior to CEU/CHB divergence is set to the maximum likelihood history from our previous Out of Africa analysis. Note that θ must be scaled to reflect the differing effective sequence length. This model reproduces the frequency spectrum as well as Model (b) (log-likelihood = -5262.0, 10 free parameters), and it yields the correct pattern of linkage disequilibrium.

6.2 Maximum likelihood parameters

Supplementary Table 6 gives the maximum likelihood parameter values for our Settlement of the New World. The corresponding *ms* demography is:

```
-n 1 1.682020 -n 2 2.424020 -n 3 4.185850 -n 4 7.942130
-es 0 4 0.522451 -ej 0 5 2
-eg 0 2 67.978337 -eg 0 3 109.406463 -eg 0 4 147.474095
-ema 0 5 x 0 0 0 x 0 x 3.960400 0 x 0 3.960400 x 0 x 0 0 0 x x x x x x x
-ej 0.029475 4 3
-ema 0.029475 5 x 0.881098 0.561966 x x 0.881098 x 3.960400 x x 0.561966
3.960400 x x x x x x x x x x x x x x
-ej 0.036114 3 2 -en 0.036114 2 0.287184
-ema 0.036114 5 x 7.293140 x x x 7.293140 x x x x x x x x x x x x x x x x
x x x
-ej 0.197963 2 1 -en 0.303500 1 1
```

Note that this command is complicated by the need to model admixture in MXL by generating a 5th population that exists for 0 time.

6.3 Parametric bootstrap

The parametric bootstrap results for our New World model are shown in Supplementary Figure 14. Note that there does appear to be some slight bias in our inference of the growth rate, r_{MX}, of the MXL population. This is because our calculation of the frequency spectrum is slightly biased in evaluation of the number of singletons private to MXL. Increasing the

number of grid points G used in the evaluation would help eliminate this bias, at the cost of an increase in computation time.

6.3.1 Parameter correlations

Supplementary Figure 15 shows the correlation between conventional bootstrap values between pairs of parameters.

Supplementary Figure 16 plots the three most correlated parameter combinations. The correlation between MXL initial population size N_{MX0} and growth rate r_{MX} is particular strong. These likely represent combinations of parameters with generate the appropriate YRI and CHB divergence.

6.4 Comparison with Out of Africa model parameters

For those inferred parameter values that are shared between our Out of Africa and Settlement of the New World Model analyses, Figure 17 compares the distributions of conventional bootstrap estimates. Importantly, all confidence intervals overlap substantially.

6.5 Admixture variability

Individuals in admixed populations mary vary considerably in their ancestry. In particular, previous analyses of Mexican-Americans have found a wide range of ancestry [14]. Our model adopts a single population-level admixture proportion, and it is important that we understand the degree to varying individual ancestry affects our results.

To roughly estimate individual ancestry proportions, we adopt a maximum-likelihood approach [15] similar to the Bayesian approach used in *structure* [16]. We consider only the European and East Asian ancestry of Mexican individuals, in order to compare with our three-population simulations. This is a rather crude approximation to the possibly very complex ancestry of these individuals, but it nevertheless provides useful guidance for our simulations.

Define q_i to be European ancestry proportion of Mexican individual i. In this rough analysis, $1-q_i$ is then that individual's East Asian ancestry proportion. For that individual, the probability of the observing the pair of alleles $x_l = (\alpha, \beta)$ at locus l is:

$$P\big(x_l = (\alpha,\beta)\big) = 2\big(\mathcal{E}_{l\alpha}q_i + \mathcal{A}_{l\alpha}(1-q_i)\big)\big(\mathcal{E}_{l\beta}q_i + \mathcal{A}_{l\beta}(1-q_i)\big). \quad \text{(S16)}$$

Here $\mathcal{E}_{l\alpha}$ is the frequency of allele α at locus l in the European population. Similarly, $\mathcal{A}_{l\alpha}$ is the frequency of allele α at locus l in the East Asian population. We simply estimate $\mathcal{E}_{l\alpha}$ and $\mathcal{A}_{l\alpha}$ by the frequencies in our CEU and CHB samples, respectively. To estimate the ancestry proportions q_i, we maximize the composite likelihood formed by multiplying the probability in Equation S16 over all loci.

The histogram in Supplementary Figure 18 shows the European admixture proportions inferred for the 22 individuals in our Mexican-American sample. The green curve shows the distribution of admixture proportions inferred from parametric bootstrap simulations of our

demographic model. These simulations were performed with $f_{MX} = 0.47$, indicated by the dashed green line. As expected, we see that our admixture inference procedure overestimates the CEU contribution, because we have no Native American samples. Also, we see that the distribution of ancestry proportions arising from our simulations is narrower than seen in the real data. In particular, there are a few individuals in the data with unusually little European ancestry.

To roughly approximate the distribution of ancestry seen in the data, we perform simulations in which 3 of our 22 individuals have 5% European ancestry, 16 have 47%, and 3 have 60%. [1] The resulting distribution of inferred ancestries is shown by the red curve in Supplementary Figure 18. This distribution captures the individuals with low-European ancestry in the real data, and is overall perhaps somewhat wider.

To test our method in the presence of an extremely wide distribution of individual ancestry, we consider a scenario in which we have 1 individual each of European ancestry proportion $\{0, 5\%, 10\% \ldots\}$. To have 22 individuals as in our data, we simulate with two individuals of European ancestry proportion 50%.[2] The resulting distribution of inferred ancestries is shown by the cyan curve in Supplementary Figure 18. As expected, the distribution of inferred ancestry proportions is very wide.

We fit our CEU/CHB/MXL model to 100 data sets each simulated with our two distribution of MXL ancestry. Supplementary Figure 19 compares the resulting parametric bootstrap parameter distributions with those from our single-ancestry-proportion simulations. The differences in f_{MX} are expected and correctly reflect the average ancestry of

[1] In *ms* parlance, the demograhpic history used was: -I 6 0 44 24 6 32 6 -n 1 1.682020 -n 2 2.424020 -n 3 4.185850 -n 4 7.942130 -es 0 4 0.950000 -es 0 5 0.530000 -es 0 6 0.400000 -ej 0 9 2 -ej 0 8 2 -ej 0 7 2 -ej 0 5 4 -ej 0 6 4 -eg 0 2 67.978337 -eg 0 3 109.406463 -eg 0 4 147.474095 -ema 0 9 x 0 0 0 x x x x x 0 x 3.9604 0 x x x x x 0 3.9604 x 0 x x x x x 0 0 0 x x x x x x x x x x x x x x x x x x x x x x x x x x x x x x x x x x x x x x x x x x x x x x x x x x x x x -ej 0.029475 4 3 -ema 0.029475 9 x 0.881098 0.561966 x x x x x x 0.881098 x 3.9604 x x x x x x 0.561966 3.9604 x x x x x x x x x x x x x x x x x x x x x x x x x x x x x x x x x x x x x x x x x x x x x x x x x x x x x x x x x x x x x -ej 0.036114 3 2 -en 0.036114 2 0.287184 -ema 0.036114 9 x 7.29314 x x x x x x x 7.29314 x x x x x x x x x x x x x x x x x x x x x x x x x x x x x x x x x x x x x x x x x x x x x x x x x x x x x x x x x x x x x x x x x x -ej 0.197963 2 1 -en 0.303500 1 1.

[2] The ms command terms handling this admixture distribution are -I 24 0 44 24 2 2 2 2 2 2 2 2 2 2 4 2 2 2 2 2 2 2 2 2 2 -n 1 1.682020 -n 2 2.424020 -n 3 4.185850 -n 4 7.942130 -es 0 4 1.000000 -es 0 5 0.950000 -es 0 6 0.900000 -es 0 7 0.850000 -es 0 8 0.800000 -es 0 9 0.750000 -es 0 10 0.700000 -es 0 11 0.650000 -es 0 12 0.600000 -es 0 13 0.550000 -es 0 14 0.500000 -es 0 15 0.450000 -es 0 16 0.400000 -es 0 17 0.350000 -es 0 18 0.300000 -es 0 19 0.250000 -es 0 20 0.200000 -es 0 21 0.150000 -es 0 22 0.100000 -es 0 23 0.050000 -es 0 24 0.000000 -ej 0 45 2 -ej 0 44 2 -ej 0 43 2 -ej 0 42 2 -ej 0 41 2 -ej 0 40 2 -ej 0 39 2 -ej 0 38 2 -ej 0 37 2 -ej 0 36 2 -ej 0 35 2 -ej 0 34 2 -ej 0 33 2 -ej 0 32 2 -ej 0 31 2 -ej 0 30 2 -ej 0 29 2 -ej 0 28 2 -ej 0 27 2 -ej 0 26 2 -ej 0 25 2 -ej 0 5 4 -ej 0 6 4 -ej 0 7 4 -ej 0 8 4 -ej 0 9 4 -ej 0 10 4 -ej 0 11 4 -ej 0 12 4 -ej 0 13 4 -ej 0 14 4 -ej 0 15 4 -ej 0 16 4 -ej 0 17 4 -ej 0 18 4 -ej 0 19 4 -ej 0 20 4 -ej 0 21 4 -ej 0 22 4 -ej 0 23 4 -ej 0 24 4.

Supplementary Table 7: P-values of rare frequency spectrum entries. The tables record the fraction of parametric bootstrap simulations yielding larger proportion of mutations in a given frequency class than observed in the data.

CHB				
2	0.13	0.32	0.63	
1	0.75	0.11	0.45	
0	—	0.02	0.59	
	0	1	2	MXL

(a) Sample CEU frequency 0

CHB				
2	0.79	0.62	0.61	
1	0.95	0.84	0.26	
0	1.00	0.77	0.65	
	0	1	2	MXL

(b) Sample CEU frequency 1

CHB				
2	0.38	0.32	0.61	
1	0.90	0.52	0.04	
0	0.94	1.00	0.94	
	0	1	2	MXL

(c) Sample CEU frequency 2

individuals in each simulation. Agreement between the distributions is outstanding. Our demographic inferences are very robust to a distribution of ancestry in the MXL individuals. Remarkably, variable ancestry does not even effect our power, as evidenced by the fact that the widths of all the parameter distributions are identical.

6.6 Rare alleles

As in section 5.6, it may be of interest specifically how well our model including MXL reproduces the distribution of shared rare alleles. Supplementary Table 7. In this case, it appears that our model may be somewhat overestimating the proportion of alleles that are observed to be absent or at very low frequency in CHB.

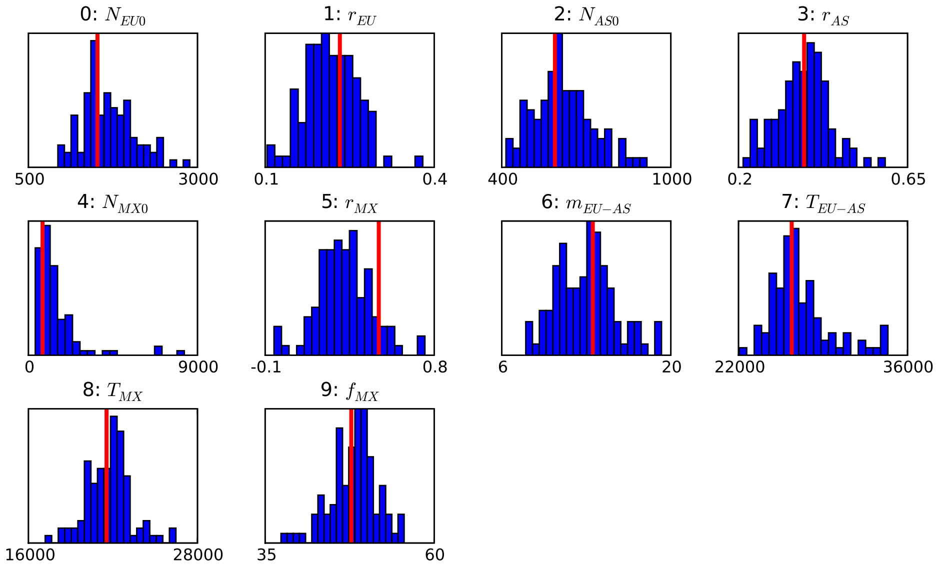

(a)

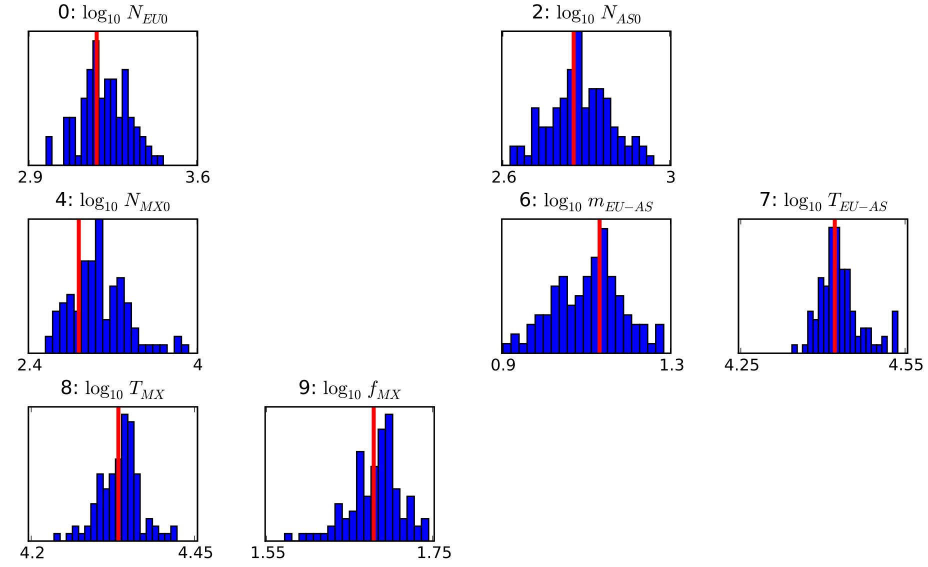

(b)

Supplementary Figure 14: Settlement of New World model parametric bootstrap results.

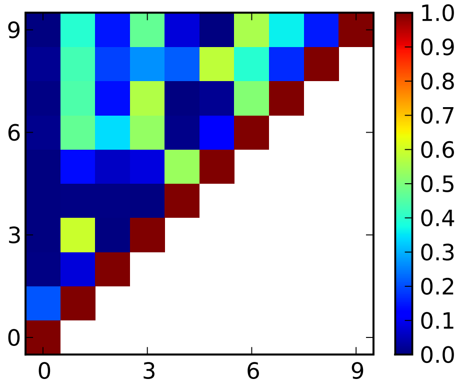

Supplementary Figure 15: Correlation (r^2) between bootstrap parameter values for Settlement of New World analysis. Parameters are indexed as in Supplementary Figure 14.

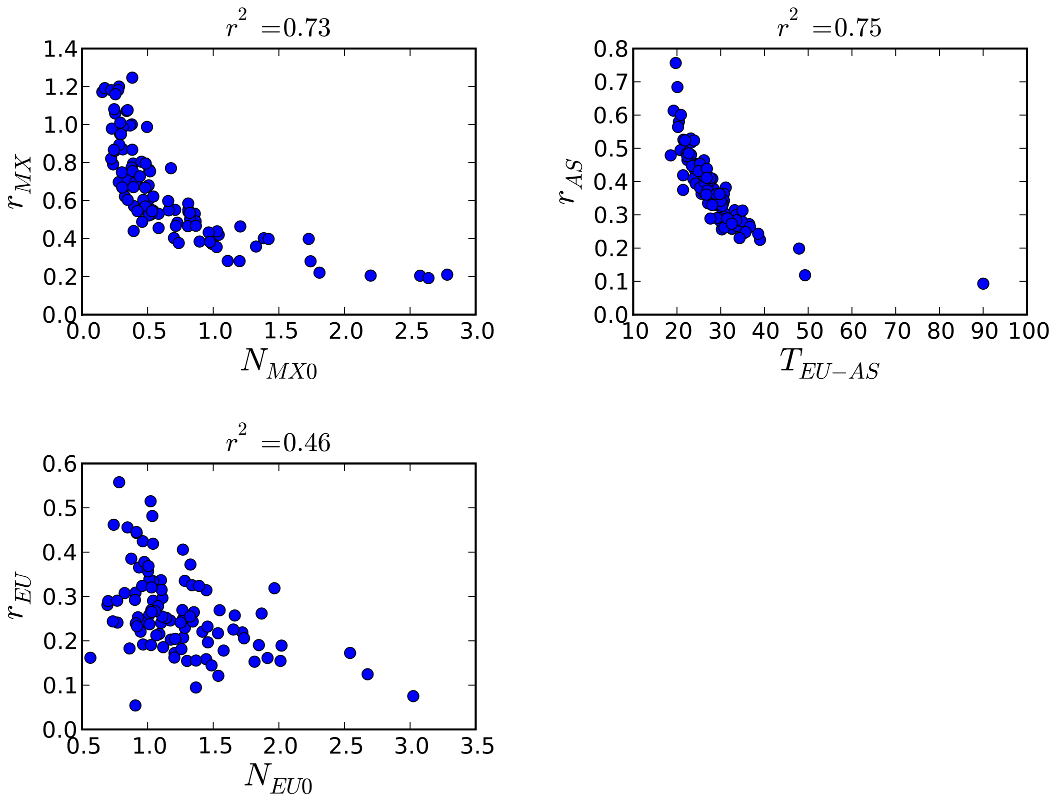

Supplementary Figure 16: Plotted are conventional bootstrap values of the three most correlated parameter pairs. (For plotting N_{MX0}, T_{EU-AS} and N_{EU0} have been divided by one thousand.)

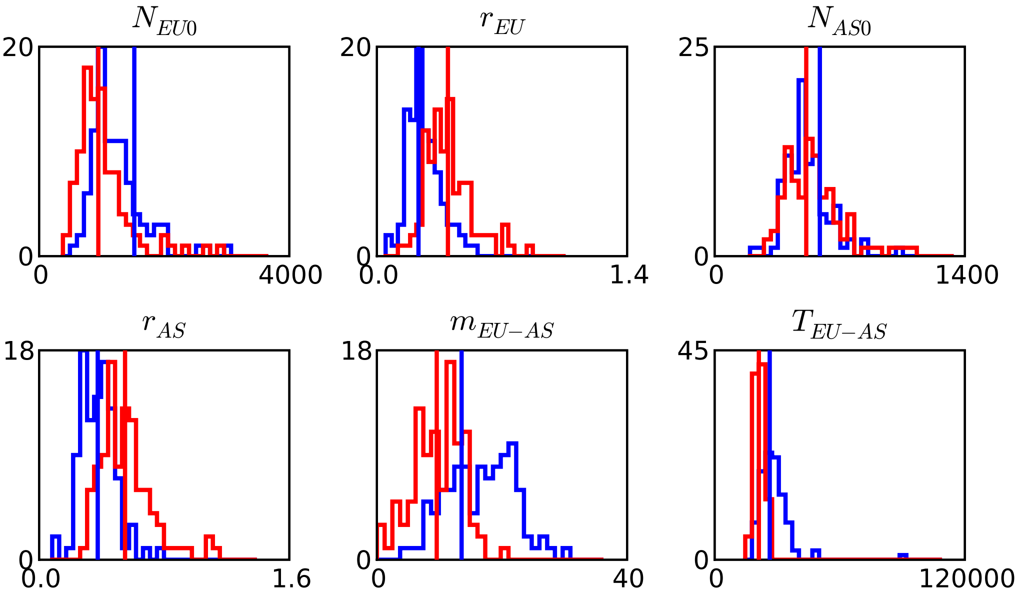

Supplementary Figure 17: Comparison of parameter inferences in Out of Africa and New World models. The New World values are in blue, while the Out of Africa are red. The maximum-likelihood values are indicated by vertical lines.

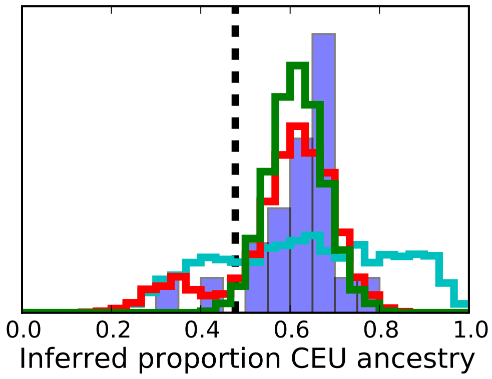

Supplementary Figure 18: Distribution of inferred European ancestry proportion for real data, and simulations with varying degrees of individual ancestry variability. Histogram is the real data. The green, red, and cyan curves are, respectively, simulations with only a single admixture proportion, with a simple distribution of admixture proportions chosen to mimic the real data, and with an extremely wide distribution. Note that these are only crude estimates of European ancestry proportion, because our data lack Native American samples. This is emphasized by the dashed black line, which shows the European admixture proportion used in the single proportion simulations.

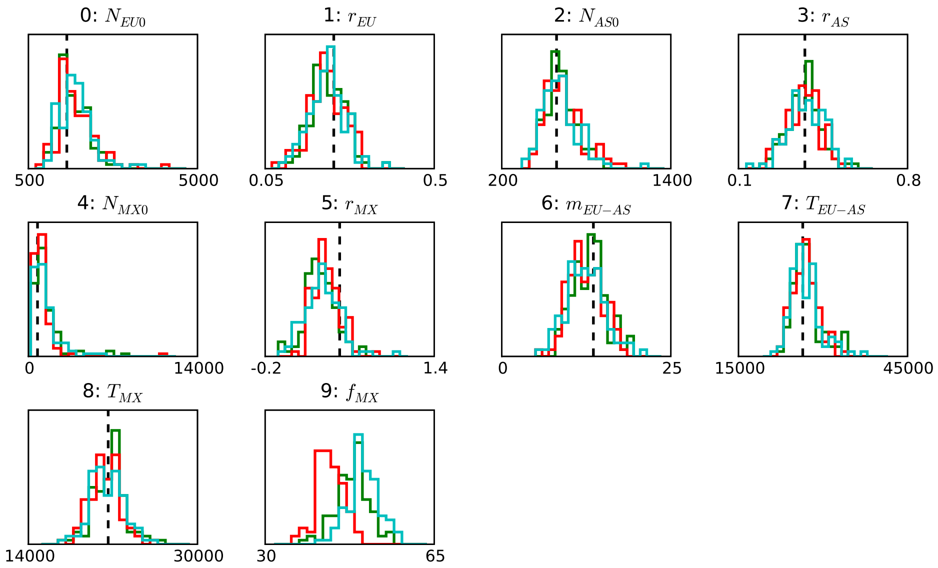

Supplementary Figure 19: Comparison of parametric bootstrap results with and without variable MXL ancestry. Black dashed lines indicate parameter values used in the simulations. The blue curve shows parametric bootstrap results for simulations with only a single Mexican admixture proportion. The red curve shows parametric bootstrap results with a distribution of Mexican admixture proportions chosen to mimic that seen in the data (see Supplementary Figure 18). The cyan curve results from an extremely wide distribution of ancestries. The excellent agreement between the distributions suggests that our inference procedure is very robust to variable ancestry in Mexican-American individuals.

7 Nonsynonymous variation

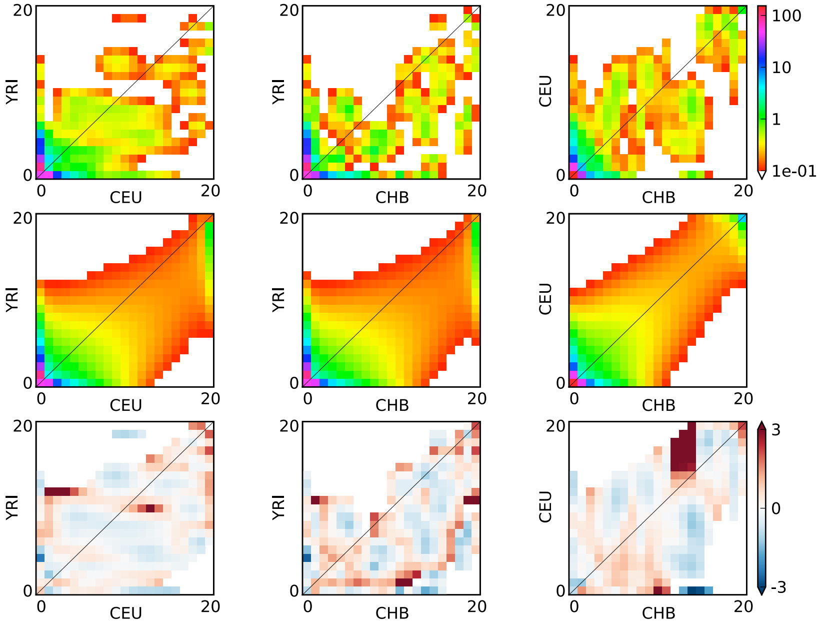

(a)

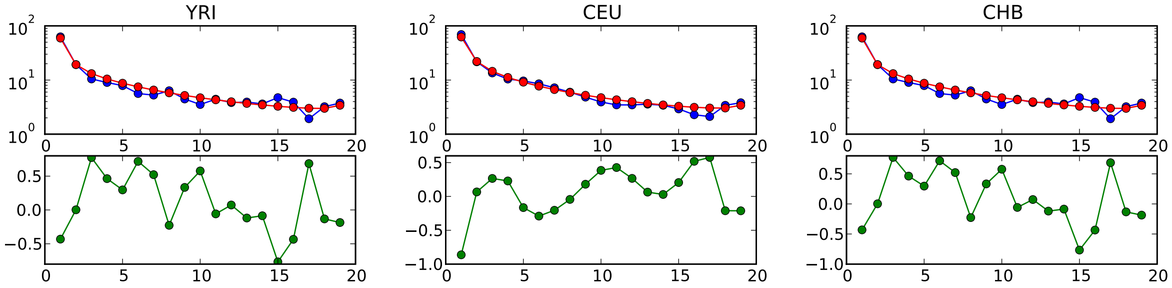

(b) (c) (d)

Supplementary Figure 20: Detailed nonsynonymous SNP comparison. Showed are comparisons of the two- and one- dimensional marginal spectra from the data with the model. Agreement is overall reasonable, although the data is too sparse for quantitative comparison of the entire AFS.

References

[1] Press WH, Teukolsky SA, Vetterling WT, Flannery BP (2007) Numerical Recipes: The Art of Scientific Computing. Cambridge University Press, 3rd edition.

[2] Chang JS, Cooper G (1970) A practical difference scheme for Fokker-Planck equations. J Comput Phys 6: 1–16.

[3] Kimura M (1964) Diffusion models in population genetics. J Appl Probab 1: 177–232.

[4] Evans SN, Shvets Y, Slatkin M (2007) Non-equilibrium theory of the allele frequency spectrum. Theor Popul Biol 71: 109–119.

[5] Hernandez RD, Williamson SH, Bustamante CD (2007) Context dependence, ancestral misidentification, and spurious signatures of natural selection. Mol Biol Evol 24: 1792–1800.

[6] Kent WJ (2002) BLAT—The BLAST-like alignment tool. Genome Res 12: 656–664.

[7] Marth GT, Czabarka E, Murvai J, Sherry ST (2004) The allele frequency spectrum in genome-wide human variation data reveals signals of differential demographic history in three large world populations. Genetics 166: 351–372.

[8] Stephens M, Donnelly P (2003) A comparison of bayesian methods for haplotype reconstruction from population genotype data. Am J Hum Genet 73: 1162–1169.

[9] Kumar S, Filipski A, Swarna V, Walker A, Hedges SB (2005) Placing confidence limits on the molecular age of the human-chimpanzee divergence. Proc Natl Acad Sci U S A 102: 18842–18847.

[10] Hudson RR (2002) Generating samples under a Wright-Fisher neutral model of genetic variation. Bioinformatics 18: 337–338.

[11] International HapMap Consortium, Frazer KA, Ballinger DG, Cox DR, Hinds DA, et al. (2007) A second generation human haplotype map of over 3.1 million SNPs. Nature 449: 851–861.

[12] Waterfall JJ, Casey FP, Gutenkunst RN, Brown KS, Myers CR, et al. (2006) Sloppy-model universality class and the Vandermonde matrix. Phys Rev Lett 97: 150601.

[13] Myers S, Fefferman C, Patterson N (2008) Can one learn history from the allelic spectrum? Theor Popul Biol 73: 342–348.

[14] Price AL, Patterson N, Yu F, Cox DR, Waliszewska A, et al. (2007) A genomewide admixture map for latino populations. Am J Hum Genet 80: 1024–1036.

[15] Nielsen R, Hubisz M, Hellmann I, Torgerson D, Andrés A, et al. Darwinian and demographic forces affecting human protein coding genes. In press.

[16] Pritchard JK, Stephens M, Donnelly P (2000) Inference of population structure using multilocus genotype data. Genetics 155: 945–959.

Supplementary Table 8: Properties of genes analyzed. Recorded are the location of each gene, the number of noncoding bases sequenced, and the number of SNPs found in that sequence. Note that these are SNPs segregating in any of the EGP populations, of which we've considered subsets. The horizontal lines divide genes that were considered contiguous possibly linked blocks in our simulations with linkage. For each of these 194 blocks, the recombination rate used is reported.

Gene	Location	Noncoding sequenced	SNPs	Recomb. rate (cM/Mb)
tnfrsf4	chr1:1134785-1141404	5785	53	0.89
eno1	chr1:8842305-8863311	15246	83	0.26
angptl7	chr1:11170249-11180532	8209	56	0.05
mad2l2	chr1:11655241-11665757	9026	75	3.47
ece1	chr1:21416988-21490465	40367	234	0.99
rpa2	chr1:28088693-28115249	23201	142	0.21
cdc20	chr1:43595293-43603425	6632	21	0.04
prdx1	chr1:45747295-45761481	12662	89	0.08
cyp4b1	chr1:47035798-47059298	15169	132	0.52
gpx7	chr1:52839455-52849118	8496	53	0.31
mrpl37	chr1:54436478-54458642	17729	111	4.22
gstm3	chr1:110076140-110086554	9480	52	0.45
dclre1b	chr1:114247570-114260035	9519	38	4.74
fmo5	chr1:145123019-145164842	16491	117	0.30
mcl1	chr1:148811707-148820540	6884	26	0.09
sprr3	chr1:151238865-151243575	3822	54	0.01
hspa6	chr1:159758724-159765190	3836	74	1.09
cd3z	chr1:165665594-165756452	35760	251	5.30
fmo3	chr1:169324675-169355337	19347	161	
fmo2	chr1:169419962-169446946	24498	201	0.56
fmo4	chr1:169548369-169579838	23589	90	1.52
prdx6	chr1:171711198-171725810	12879	73	0.17
abl2	chr1:177341425-177466679	30983	166	0.04
glrx2	chr1:191330262-191343224	10690	39	0.00
sphar	chr1:227504932-227509818	4694	21	0.00
dclre1c	chr10:14988098-15038104	33128	283	1.29
sirt1	chr10:69313282-69350134	23368	135	0.06
pten	chr10:89611246-89718010	44318	171	0.04
pdlim1	chr10:96986027-97042728	40888	267	3.21
mms19l	chr10:99206900-99249416	31198	133	0.28
cyp17a1	chr10:104578288-104587866	6830	39	0.28
prdx3	chr10:120915578-120929924	12842	89	1.00
fgfr2	chr10:123226932-123349844	47179	321	1.92
mmp21	chr10:127443010-127456384	11664	68	0.47
bnip3	chr10:133629245-133647430	16068	88	0.21
cyp2e1	chr10:135188892-135204545	10388	80	1.62
muc2	chr11:1063774-1096409	18417	203	
muc5ac	chr11:1130549-1247305	43767	435	8.59
adm	chr11:10281267-10287423	5598	20	0.08
calca	chr11:14945166-14951958	5857	31	0.18
fancf	chr11:22598688-22605597	5784	36	0.26
depc1	chr11:43857038-43900374	21499	157	0.26
ddb1	chr11:60821542-60858358	24953	140	0.02
esrra	chr11:63828047-63842665	9753	44	
prdx5	chr11:63840191-63847827	6991	39	0.16
fibp	chr11:65406224-65414553	7234	40	0.02
pold4	chr11:66873631-66879563	5608	24	0.01
fadd	chr11:69724915-69731534	5992	35	0.81
ucp2	chr11:73362345-73373540	8695	45	0.85
birc2	chr11:101721227-101756502	16890	99	0.19
mmp8	chr11:102086541-102102880	14746	114	0.38
mmp12	chr11:102236716-102252838	13144	60	0.21
casp4	chr11:104317257-104346525	19430	124	
casp5	chr11:104368183-104385539	12732	96	3.07
bace1	chr11:116659883-116694031	28147	101	0.66

foxm1	chr12:2835814-2858359	13105	84	0.44
cd4	chr12:6767038-6800499	20508	124	3.67
ddit3	chr12:56195043-56201780	5857	25	0.10
txnrd1	chr12:103203105-103269990	39658	208	0.09
tuba2	chr13:18643916-18655936	10143	134	1.53
tgm1	chr14:23786446-23804157	14147	84	4.81
prkd1	chr14:29113440-29468639	117650	709	1.01
hspa2	chr14:64070670-64081266	8398	50	0.26
rad51l1	chr14:67354260-68014651	54253	286	0.32
mlh3	chr14:74551067-74589968	26770	106	0.06
ngb	chr14:76799588-76809367	8578	25	0.29
tdp1	chr14:89489997-89582627	54024	450	0.09
atxn3	chr14:91597290-91644709	33289	233	0.39
cdc42bpb	chr14:102466485-102594317	68463	555	0.37
ckb	chr14:103053810-103060893	4975	26	0.58
tjp1	chr15:27777670-27903095	64491	206	0.06
capn3	chr15:40437058-40493743	33273	259	0.41
lcmt2	chr15:41405704-41412105	4004	27	
tp53bp1	chr15:41484739-41591642	42841	155	0.06
dut	chr15:46409739-46424845	14611	73	0.13
cyp19a1	chr15:49286972-49420103	41602	235	0.77
aldh1a2	chr15:56030935-56147219	41406	297	0.21
ppib	chr15:62233067-62244406	7645	29	0.51
cyp1a1	chr15:72797088-72806750	7744	41	
csk	chr15:72859819-72884394	20901	104	0.34
blm	chr15:89059760-89160889	43775	320	1.91
abcc1	chr16:15949029-16144720	77618	548	2.36
itgal	chr16:30390443-30443977	32203	98	1.04
tgfb1i1	chr16:31390025-31398732	6253	29	0.14
mt3	chr16:55178288-55185470	6383	54	
mt2a	chr16:55198077-55202889	4315	26	1.60
mmp15	chr16:56614812-56639604	20075	127	0.67
ces2	chr16:65523966-65538512	11835	34	0.10
cdh1	chr16:67327048-67427588	33768	192	1.03
map2k4	chr17:11863715-11989045	52874	244	0.78
nos2a	chr17:23105934-23153691	40735	245	0.99
traf4	chr17:24094767-24102944	4920	17	0.05
slc6a4	chr17:25547870-25588397	32318	163	1.23
ccl5	chr17:31220679-31233116	8983	43	0.15
aoc2	chr17:38248179-38256964	5192	31	
aoc3	chr17:38254788-38265391	8311	53	0.91
epx	chr17:53623637-53638737	11958	66	
mpo	chr17:53700284-53715294	12360	59	1.04
fdxr	chr17:70369763-70382705	10034	80	0.76
flj35220	chr17:76002438-76028361	22577	218	1.17
tbxa2r	chr19:3543524-3559600	8156	55	2.61
retn	chr19:7638899-7643335	3094	23	12.40
cdc37	chr19:10360810-10376873	9676	61	0.53
prdx2	chr19:12767010-12775436	6352	20	0.13
jund	chr19:18249798-18255411	4569	31	0.21
uba52	chr19:18541857-18551267	7682	76	0.63
cyp2a6	chr19:46039745-46049329	6359	24	0.85
cyp2b6	chr19:46188528-46217135	12150	114	0.53
fosb	chr19:50661388-50672275	7981	48	0.62
fgf21	chr19:53949867-53954694	3266	26	4.08
tpo	chr2:1394275-1527510	52005	451	1.04
odc1	chr2:10495964-10507871	8829	77	2.89
osr1	chr2:19412748-19423807	10107	57	3.98
tp53i3	chr2:24151902-24163166	9080	37	0.02
xdh	chr2:31410740-31492750	49855	359	1.90
srd5a2	chr2:31601222-31661522	25841	149	0.27

rev1l	chr2:99381900-99474946	47250	215	0.04
sult1c2	chr2:108358851-108372202	11442	57	0.22
casp10	chr2:201753864-201795925	36933	181	
casp8	chr2:201804951-201862677	25564	155	0.32
xrcc5	chr2:216679600-216781263	59688	377	0.33
cdk5r2	chr2:219530783-219536873	4688	22	0.48
tuba1	chr2:219821294-219828881	5335	24	0.07
pax3	chr2:222770789-222873966	37987	238	2.11
ugt1a1	chr2:234331663-234348269	14764	102	3.88
stk25	chr2:242082485-242098503	11355	85	1.13
tgm3	chr20:2222687-2271646	27310	195	14.22
adam33	chr20:3594643-3612212	11441	98	9.72
gss	chr20:32977941-33008951	24298	109	0.33
tgm2	chr20:36188289-36228787	33377	239	1.48
plcg1	chr20:39197582-39239725	38122	173	0.06
mmp9	chr20:44068952-44080602	8530	45	0.45
ncoa3	chr20:45562610-45720991	67165	312	0.09
spo11	chr20:55336692-55354204	16068	72	0.20
chrna4	chr20:61444524-61465155	13979	127	3.14
app	chr21:26174598-26466748	76787	422	0.96
trpm2	chr21:44596008-44688231	46955	256	1.77
tbx1	chr22:18109239-18152828	40156	324	4.01
mapk1	chr22:20441947-20553900	44393	223	0.06
mmp11	chr22:22443300-22458501	10387	83	
smarcb1	chr22:22457747-22508636	29700	311	0.35
mn1	chr22:26472295-26529526	31530	217	9.42
mb	chr22:34331272-34345316	13084	83	0.47
rad18	chr3:8894583-8982157	47273	353	0.35
fancd2	chr3:10041369-10118048	43662	291	0.03
oxsr1	chr3:38180035-38273949	45330	196	0.04
cx3cr1	chr3:39278151-39298493	18139	123	2.52
cxcr6	chr3:45958001-45966776	6418	36	0.08
poln	chr4:2043284-2202575	63537	303	0.75
ugt2b4	chr4:70378553-70398209	16390	128	1.27
fgf5	chr4:81404858-81433179	25201	225	3.66
snca	chr4:90863805-90977228	39808	283	0.17
adh5	chr4:100209588-100231047	19125	155	
adh4	chr4:100263522-100285442	17459	167	
adh6	chr4:100342975-100360914	16267	61	
adh1a	chr4:100415366-100432987	14726	75	
adh1b	chr4:100446108-100463534	14564	69	
adh1c	chr4:100474697-100494926	12484	100	0.33
gab1	chr4:144476938-144612701	40223	175	0.48
anapc10	chr4:146133771-146240749	31273	128	0.01
neil3	chr4:178466441-178522793	31513	236	2.59
cyp4v2	chr4:187347686-187372899	19389	157	3.60
tert	chr5:1304982-1349971	27598	235	3.94
rad1	chr5:34939140-34956145	14622	83	0.13
sepp1	chr5:42834018-42849661	14107	69	0.10
xrcc4	chr5:82407721-82686781	76892	515	0.57
glrx	chr5:95173453-95186148	9618	72	1.99
ube2b	chr5:133732767-133757695	22770	118	0.02
hspa9b	chr5:137917554-137941006	17066	64	0.07
ctnna1	chr5:138144392-138300374	37327	178	0.04
atox1	chr5:151100578-151120117	16976	80	0.21
dusp1	chr5:172125723-172132307	4900	31	1.12
fgfr4	chr5:176445082-176458717	8382	45	0.52
mapk9	chr5:179593434-179642220	41550	270	0.44
gpx6	chr6:28578919-28593498	11224	69	
gpx5	chr6:28599802-28611651	9891	47	0.16
msh5	chr6:31813759-31842569	21092	108	
hspa1l	chr6:31883771-31891515	5083	33	

hspa1a	chr6:31889373-31895694	2453	17	
hspa1b	chr6:31901538-31907733	4013	30	0.13
fance	chr6:35527645-35544820	13668	81	0.17
gsta3	chr6:52867429-52884390	13987	93	
gsta4	chr6:52950677-52970041	14568	99	2.54
marcks	chr6:114283284-114291631	7237	24	3.17
hsf2	chr6:122760560-122797817	29209	108	0.04
vnn1	chr6:133042458-133078042	16990	141	
vnn3	chr6:133084723-133099338	13790	121	
vnn2	chr6:133104704-133122726	14925	122	3.14
nudt1	chr7:2248275-2257703	8506	96	0.68
rac1	chr7:6380405-6412143	27091	252	0.35
rpa3	chr7:7641155-7726729	39301	284	1.63
polm	chr7:44077383-44090597	11122	64	0.08
igfbp3	chr7:45916382-45929339	11936	61	5.63
por	chr7:75420622-75454411	15593	114	3.20
hspb1	chr7:75769508-75772601	2475	20	1.28
abcb4	chr7:86867353-86949645	69500	410	
abcb1	chr7:86970771-87182449	88991	468	0.41
cyp3a5	chr7:99083564-99115770	16185	76	
cyp3a4	chr7:99191632-99221666	20737	67	0.50
abp1	chr7:150178590-150191285	8699	55	2.11
slc4a2	chr7:150385719-150406496	15421	103	1.09
msr1	chr8:16008774-16094804	24853	268	3.76
scara3	chr8:27545520-27588149	37814	303	1.02
ube2v2	chr8:49082972-49138936	15669	41	0.05
terf1	chr8:74081659-74123686	29263	205	0.10
mmp16	chr8:89120172-89410308	70209	382	0.91
rrm2b	chr8:103284760-103322295	30205	164	0.45
oxr1	chr8:107739110-107833889	37475	141	0.05
cyc1	chr8:145220372-145226365	4786	26	0.07
recql4	chr8:145705653-145715945	6665	61	0.41
ifna1	chr9:21428453-21432913	3554	36	0.36
dapk1	chr9:89300614-89513612	97580	718	3.56
fbp1	chr9:96403244-96443887	30956	258	0.37
ptch	chr9:97244275-97311000	33249	175	0.87
pole3	chr9:115207883-115214172	5845	36	0.26
hspa5	chr9:127035208-127045070	7560	39	0.23
ciz1	chr9:129966204-130008434	21746	139	0.64
abl1	chr9:132577087-132753794	52704	371	0.87
rxra	chr9:136431296-136474254	32537	351	1.99
